# Analysis of the network pharmacology and the structure-activity relationship of glycyrrhizic acid and glycyrrhetinic acid

**DOI:** 10.3389/fphar.2022.1001018

**Published:** 2022-10-13

**Authors:** Qingqiang Ni, Yuxuan Gao, Xiuzhen Yang, Qingmeng Zhang, Baojian Guo, Jinxiang Han, Shaoru Chen

**Affiliations:** ^1^ Department of Hepatobiliary Surgery, Shandong Provincial Hospital Affifiliated to Shandong First Medical University, Jinan, Shandong, China; ^2^ Postdoctoral Mobile Station, Shandong University of Traditional Chinese Medicine, Jinan, Shandong, China; ^3^ Department of Basic Research, Guangzhou Laboratory, Guangzhou, Guangdong, China; ^4^ Department of Orthopaedics, Qilu Hospital of Shandong University, Jinan, Shandong, China; ^5^ Institute of New Drug Research, International Cooperative Laboratory of Traditional Chinese Medicine Modernization and Innovative Drug Development of Chinese Ministry of Education, Jinan University College of Pharmacy, Guangzhou, Guangdong, China; ^6^ Biomedical Sciences College and Shandong Medicinal Biotechnology Centre, Shandong First Medical University and Shandong Academy of Medical Sciences, Jinan, Shandong, China

**Keywords:** licorice, glycyrrhizic acid, glycyrrhetinic acid, network pharmacology, structure-activity relationship, structure modification

## Abstract

Licorice, a herbal product derived from the root of *Glycyrrhiza* species, has been used as a sweetening agent and traditional herbal medicine for hundreds of years. Glycyrrhizic acid (GL) and glycyrrhetinic acid (GA) are the most important active ingredients in licorice. Both GL and GA have pharmacological effects against tumors, inflammation, viral infection, liver diseases, neurological diseases, and metabolic diseases. However, they also exhibit differences. KEGG analysis indicated that licorice is involved in neuroactive ligand‒receptor interactions, while 18β-GA is mostly involved in arrhythmogenic right ventricular cardiomyopathy. In this article, we comprehensively review the therapeutic potential of GL and GA by focusing on their pharmacological effects and working mechanisms. We systemically examine the structure-activity relationship of GL, GA and their isomers. Based on the various pharmacological activities of GL, GA and their isomers, we propose further development of structural derivatives of GA after chemical structure modification, with less cytotoxicity but higher targeting specificity. More research is needed on the clinical applications of licorice and its active ingredients.

## Introduction

**FIGURE 1:**
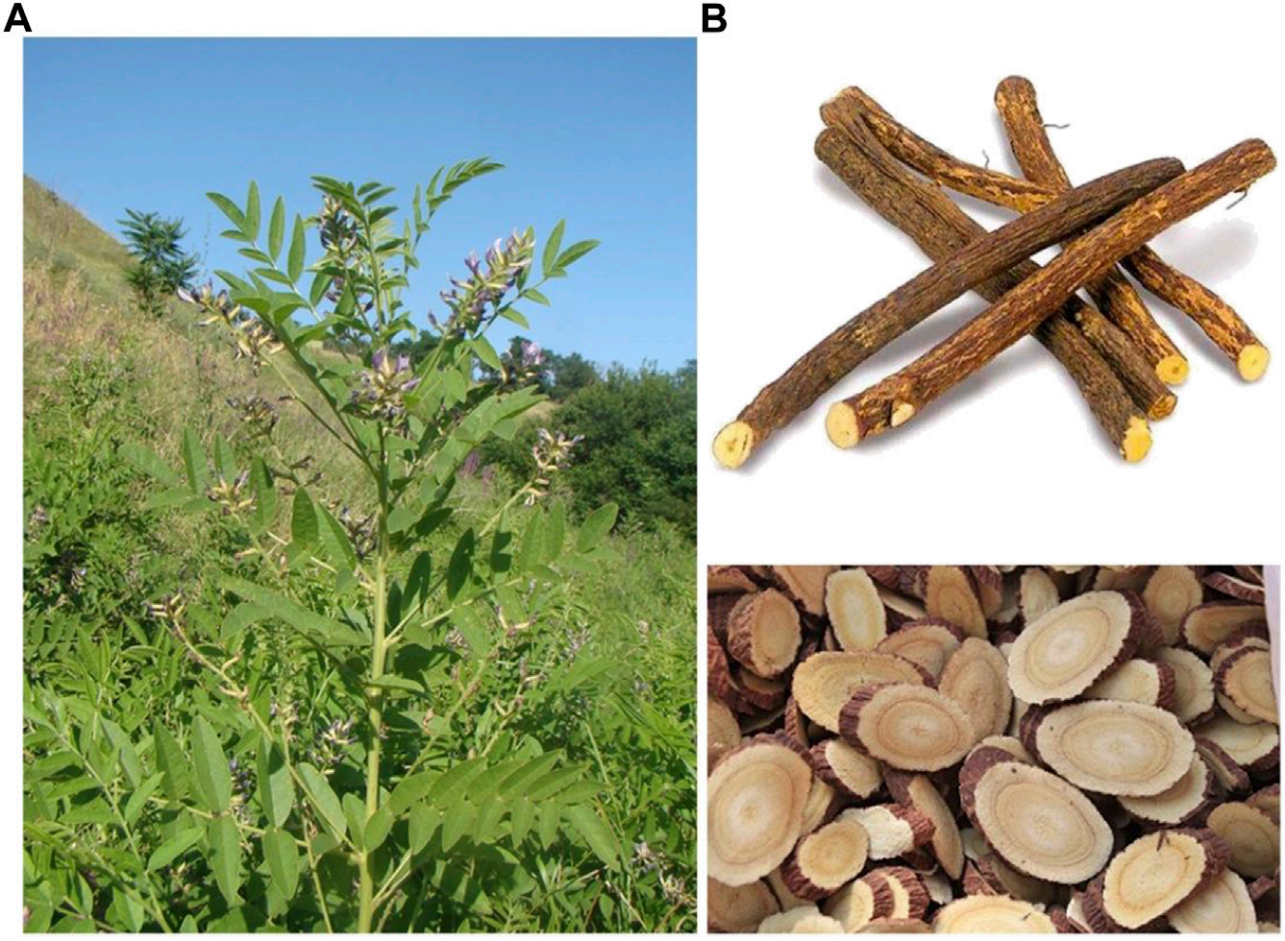
Pictures of licorice. **(A)** *Glycyrrhiza*; **(B)** Licorice.

Gancao (licorice) is a perennial herb with multiple varieties and a popular herbal medicine derived from dry roots and rhizomes of *G. glabra*, *G. inflata*, and *G. uralensis* (Zhang and Ye, 2009). Licorice has been also widely used for hundreds of years in Eastern and Western countries, including China, Japan, India, Russia, Spain, and the United Kingdom (Wang et al., 2020). *Glycyrrhiza* is a dwarf shrub with oval leaflets, white or purple flower clusters, and flat pods, as shown in Figure 1A. The genus name *Glycyrrhiza* is derived from the ancient Greek words *glykos* and *rhiza*, meaning sweet and root, which were later latinized to liquiritia, and this word eventually evolved into *licorice* (Karaaslan and Dalgic, 2014).

Licorice, an important commercial product, can be used to treat asthma and dry cough and prevent thirst (Yu et al., 2018; Isbrucker and Burdock, 2006). Licorice, one of the oldest and most commonly used traditional Chinese medicines (Figure 1B), can be used to cure gastric ulcers, cough, bronchitis, heartburn, eczema, and inflammation (Yu et al., 2018).

Over the past century, a large number of components have been isolated from licorice. Water-soluble biologically active compounds account for 40% to 50% of the total weight of licorice dry matter. Licorice contains major biologically active ingredients, such as flavonoids, saponins, sterols, starch, amino acids, gums, and essential oils, including the active compounds glycyrrhizin, glabridin, liquiritin, glycyrrhizic acid (GL), and glycyrrhetinic acid (GA) (El-Saber Batiha et al., 2020). Triterpene saponins are also major components in licorice. Recently, approximately 77 kinds of triterpene saponins have been isolated from licorice (Li et al., 2020).

## Methods

### Gene target acquisition and screening

For a comprehensive determination of licorice, establishing licorice-, GA (18β-GA and 18α-GA)- and GL-target interaction profiles is a critical step for systems biology analysis. Recently, the Traditional Chinese Medicine Systems Pharmacology Database and Analysis Platform (TCMSP) and Integrative Pharmacology-based Research Platform of Traditional Chinese Medicine (TCMIP) were used to acquire potential targets of licorice, GA (18β-GA and 18α-GA) and GL. In our article, we use the TCMSP database to acquire potential targets of licorice, GA (18β-GA and 18α-GA) and GL. Then, databases such as GeneCards, OMIM, pharmGkb, and TTD are used to acquire potential targets involved in inflammation.

The target information found in these five databases is complementary, and their combination could provide relatively comprehensive compound-target interactions. In this work, the targets of licorice, GA (18β-GA and 18α-GA), GL, and inflammation were examined separately using direct text mining of TCMSP, GeneCards, OMIM, pharmGkb, and TTD, together with their chemical names and inflammatory diseases as keywords. The targets of licorice, GA (18β-GA and 18α-GA), and GL from TCMSP, GeneCards, OMIM, pharmGkb, and TTD with interaction counts less than the median were excluded. All acquired targets of licorice, GL, and GA (18β-GA and 18α-GA) were limited to *Homo sapiens* and mapped to UniProt for correction to remove redundant and erroneous targets. Finally choose the compound with higher OB/DL value together with licorice for further Kyoto Encyclopedia of Genes and Genomes pathway analysis (KEGG) as well as Protein-Protein interaction analysis.

### Kyoto Encyclopedia of Genes and Genomes pathway analysis

KEGG pathway enrichment and analysis are performed on ClueGo integrated with the KEGG database (updated on 1 May 2022). The procedures are similar to the immune system process for GO terms analysis, described briefly as following:

(1) Import the targets of licorice and 18β-GA to ClueGo separately, as represented by different colors;
(2) Set visual style as “cluster,” and set the statistical method as a two-sided hypergeometric test and Bonferroni step-down adjustment, and only pathways with *p* value less than 0.05 are shown;
(3) Begin the analysis: download the protein-pathway interaction information into Excel format for analysis. According to the KEGG database, the pathways are clustered into the following categories: (A) metabolism, (B) genetic information processing, (C) environmental information processing, (D) cellular processes, (E) organismal systems, and (F) human diseases. Finally, the top thirty protein–pathways on licorice related to human inflammatory diseases and nine protein-pathways on 18β-GA related to human inflammatory diseases are extracted and shown.

### Protein‒Protein interaction analysis

Hub targets are identified by taking the following steps:

(1) Combine the targets of licorice and 18β-GA then remove the duplicates;
(2) Map them into the TTD website, choose the “inflammation diseases” genes database for comparison, and select the overlapping targets for the next analysis;
(3) Map select targets into STRING (version 11.5) to perform the PPI analysis; set the cutoff degree of PPI to high confidence (0.400), and download the PPI information the TSV file format;
(4) Import the file into Cytoscape software (version 3.8.0) to analyze the topological parameters of the interactions and select the hub targets whose node degrees are greater than the median value. After these steps, STRING and Cytoscape are used to construct and analyze the PPI network of the hub targets. In the constructed networks, the targets are represented by nodes, while the interactions among them are represented by edges.

## Results

**FIGURE 2:**
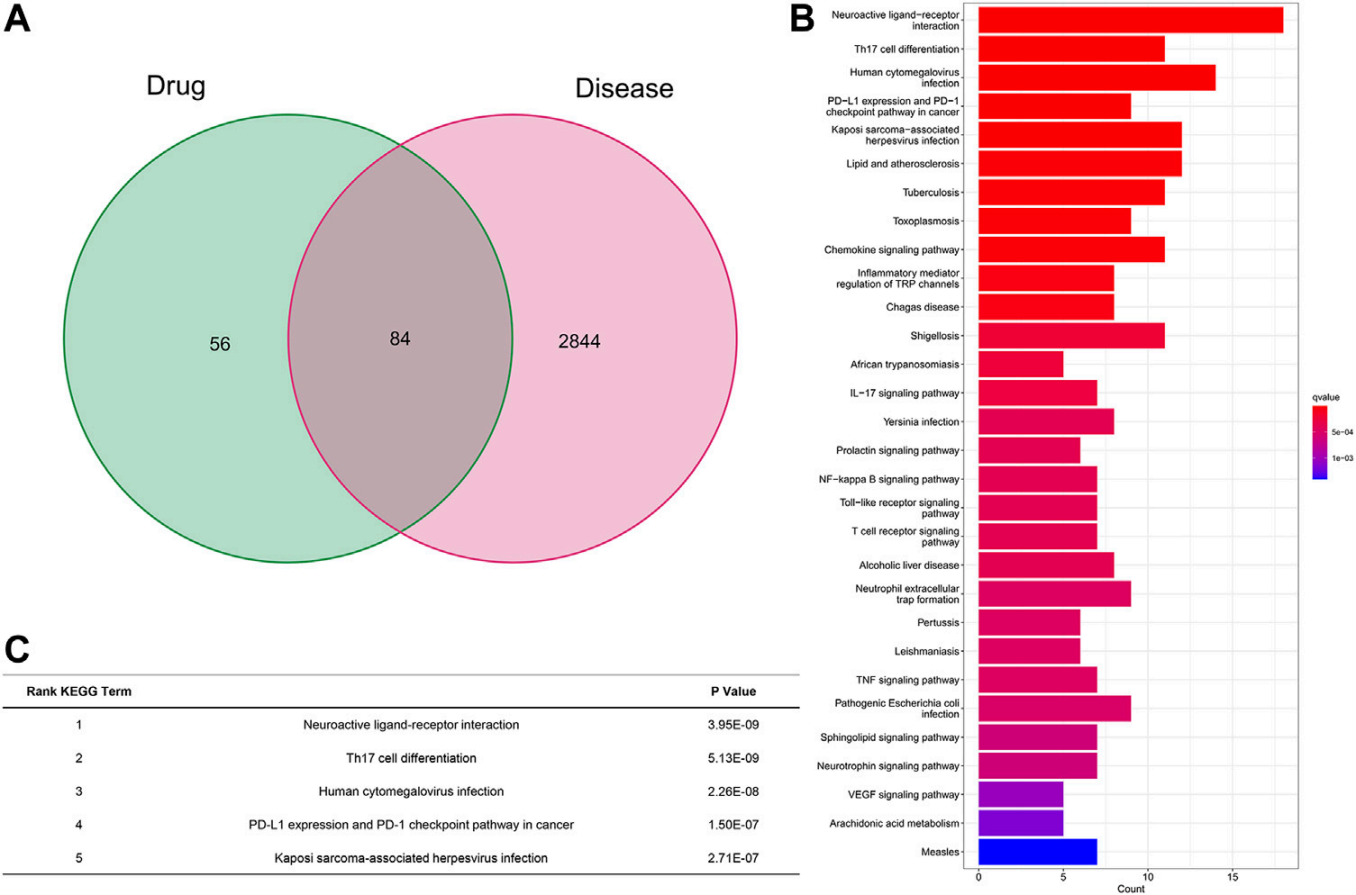
KEGG pathway analysis of licorice among 84 gene sets. **(A)** Summary of significant, differentially expressed genes. **(B)** KEGG pathway analysis of the 84 genes differentially targeted by licorice related to inflammation. **(C)** Most important interaction between licorice and inflammation according to KEGG pathway analysis.

After all of the databases were analyzed and searched, based on the oral bioavailability value (OB value) and drug-like property (DL value) we searched and analyzed from all the databases, we got the OB and DL value of GA and GL in Table 1. Then we choose licorice and 18β-GA (OB% = 22.05, DL = 0.74) for further analysis for the OB and DL value higher. We found that the neuroactive ligand receptor remained the most significant pathway, even after omitting genes differentially expressed in licorice and inflammation based on the Genecard, OMIM, Pharmgkb, and TTD database analyses (Figure 2A). KEGG analyses of all genes were significantly differently expressed between licorice and inflammation, which indicated that neuroactive ligand‒receptor interacted most closely to licorice (*q* value = 3.08E-07, *p* value = 3.95E-09, *p*. adjust = 5.47E-07). Additionally, licorice also interacted with Th17-cell differentiation (*q* value = 3.08E-07, *p* value = 5.13E-09, *p*. adjust = 5.47E-07), human cytomegalovirus infection (*q* value = 9.03E-07, *p* value = 2.26E-08, *p*. adjust = 1.60E-06), PD-L1 and PD-1 checkpoint pathways in cancer (*q* value = 4.50E-06, *p* value = 1.50E-07, *p*. adjust = 7.98E-06), Kaposi sarcoma-associated herpesvirus infection signaling pathway (*q* value = 6.52E-06, *p* value = 2.71E-07, *p* adjust = 1.16E-05), and so on (Figures 2B,C). OB value and DL value of major ingredients-GA and GL in licorice (Table 1).

**TABLE 1:** OB value and DL value of major ingredients-GA and GL in licorice.

Name	OB(%) value	DL value
GL	19.62	0.11
18-β-GA	22.05	0.74
18-α-GA	17.41	0.74

The ingredients of licorice, including flavonoids, saponins, sterols, starch, amino acids, gums, and essential oils, can interact with the 26 key proteins involved in inflammatory diseases analyzed by STRING (Table 2). From the results of the analyses found in the databases and in the literature, we found that licorice as well as the specific important compounds of licorice (glycyrrhizin, glabridin, liquiritin, and so on) can interact with different proteins (Table 2). Licorice and its important ingredients play important roles in inhibiting inflammation. They can exert strong inhibition of inflammation through different targeting proteins (Table 2). The proteins that interact with the ingredients in licorice are shown in Table 2 (Chen C. et al., 2018; Oh et al., 2021; Zhou et al., 2021).

**TABLE 2:** Proteins Interacting with Ingredients in Licorice.

Gene Names	Full name	Compounds in licorice the protein interacts with
IL1β	Interleukin 1 beta	Liquiritin, glycyrrhizin
CD274	Programmed cell death ligand 1 (PDL1)	Glycyrrhizin
CRP	C-reactive protein	Glabridin
KIT	Proto-oncogene receptor tyrosine kinase	Licorice
HSP90AA1	Heat shock protein 90 alpha family class A member 1	Licorice
CCR2	C-C motif chemokine receptor 2	Glycyrrhizin
STAT3	Signal transducer and activator of transcription 3	Glycyrrhizin, 18β-glycyrrhetinic acid, isoliquiritigenin, glycyrrhizic acid, licochalcone A
ALOX5	Arachidonate 5-lipoxygenase	Null
SRC	Proto-oncogene tyrosine-protein kinase	Isoliquiritigenin
ICAM1	Intercellular adhesion molecule 1	Glycyrrhetinic acid,
NR3C1	Nuclear receptor subfamily 3 group C member 1	Null
TLR9	Toll-like Receptor 9	Glycyrrhizin
FPR1	Formyl peptide receptor 1	Null
CCR5	C-C chemokine receptor type 5	Null
SELL	Selectin L	Null
MAPK1	Mitogen-activated protein kinase 1	Glycyrrhizin
Jak3	Janus kinase 3	Null
MAPK14	Mitogen-activated protein kinase 14	Null
MPO	Myeloperoxidase	Licorice, glycyrrhizin
NOS2	Nitric oxide synthase 2	Glycyrrhizin, licorice
PTGS2	Prostaglandin-endoperoxide synthase 2	Licorice
LCK	Proto-oncogene	Null
PLA2G1B	Phospholipase A2	Glycyrrhizin
ABL1	Proto-oncogene 1, non-receptor tyrosine kinase	Null
mTOR	Mechanistic target of rapamycin kinase	Glycyrrhizin
PPARG	Peroxisome proliferator-activated receptor gamma	Licorice

As the main active ingredient in licorice, GL is the most abundant constituent of triterpenoid saponins, accounting for 10% to 25% of licorice. GL is made up of one molecule of GA and two molecules of glucuronic acid (Wei et al., 2018; Liang et al., 2019). GL and GA both have 18α and 18β stereoisomer forms. The different stereoisomer forms of GL and GA exhibit different activities (Sun Y. et al., 2018). Based on the OB and DL value, we hypothesis that 18β stereoisomer form should be a more activate form (Table 1).

Over the past few decades, the active compound GL has been widely used in many countries in clinical contexts. Magnesium isoglycyrrhizinate injection (MgIG, Chia Tai Tianqing, Pharmaceutical Co., ltd, China) is one kind of GL preparation, which is used to treat chronic liver diseases and pulmonary fibrosis in China (Zhou et al., 2022). Stronger neo-minophagen C (SNMC, Minophagen Pharmaceutical, Tokyo, Japan) is a compound GL tablet, used for treating chronic hepatic diseases in Japan for a long time (Mori et al., 1990). Other drugs also include GL phytosome, used in Italy, and the Russian drug formulation phosphogliv, produced with GL (Ochi et al., 2016). In addition, licorice is also used in food, cosmetics, and tobacco around the world. Therefore, a review focusing on the pharmacological effects of GA and GL would be very useful for identifying further therapeutic effects and promoting its development.

### Pharmacological activities of glycyrrhizic acid

GL is a main bioactive component of licorice. It exhibits a variety of pharmacological activities, including antiviral activity, anti-inflammatory activity, antitumor activity, antiallergic activity, antihyperglycemia (diabetes), and hyperlipidemia activity. In addition, it also has hepatoprotective effects, neuroprotection, renal protection, tyrosinase and thrombin inhibitory activity, steroid hormone, and estrogenic activity (Sun et al., 2019).

#### Antiviral activity

GL or its medicinal preparation shows strong inhibition of the replication of several viruses *in vitro*: Epstein‒Barr virus (EBV), herpes simplex virus (HSV), hepatitis A/B/C virus (HAV, HBV, HCV), human cytomegalovirus (CMV), human immunodeficiency virus (HIV), influenza virus (H3N2), SARS coronavirus, influenza A virus (IAV), influenza A virus (H5N1), and varicella-zoster virus (VZV) all show good sensitivity to GL *in vitro* (Yao et al., 2020; Baltina and Kondratenko, 2021). The effect of GL plus vitamin C against pneumonia caused by infection with SARS-CoV-2 was tested in a clinical trial (http://www.chictr.org.cn/). The combination of GL, vitamin C, and curcumin also exhibited potential against novel coronavirus infection through an innate immune response by acting on NOD-like and Toll-like signaling pathways, PI3K/AKT/NF-κB and MAPK signaling pathways under bioinformatics analysis (Chen et al., 2020).

The early research of Wolkerstorfer et al. (2009) clarified the antiviral effects and mechanisms of GL *in vitro*. They drew clear conclusions on the antiviral effects of GL against human lung epithelial cells and fibroblasts infected by different subtype influenza A viruses: GL can inhibit viral uptake by interfering with cell membranes and ultimately reducing endocytosis.

In another study, it was found that GL, as a new EBV replication inhibitor, exhibited a different working mechanism from existing nucleoside analogs. It could inhibit EBV DNA replication and viral antigen synthesis in Raji cells in a dose-dependent manner (Lin, 2003; Bentz et al., 2019). GL can also selectively inhibit the proliferation of lymphocytes infected with Kaposi’s sarcoma-associated herpesvirus (KSHV). GL can disrupt RNA polymerase II (RNAPII) complex formation, which accumulates at the CTCF-cohesive binding site of the first intron during transcription and then inhibits RNA virus replication. GL and its derivatives can also inhibit Zika virus replication through a strong interaction with the active site pocket of NS5 MTase (Kang and Lieberman, 2011; Baltina et al., 2021).

The anti-influenza activity of GL has been demonstrated in mice. It is found that GL plus glutamine-tryptophan treatment exhibits an inhibitory effect against the replication of oseltamivir-resistant virus A/Vladivostok/2/09 (H1N1), which can induce lethal influenza in BALB/c mice and increase their survival rate (Smirnov et al., 2013; Smirnov et al., 2014). GL and its derivatives also reduced the infectivity of Dengue virus in *Vero* E6 cells (Baltina and Kondratenko, 2021). However, the details of the mechanism by which GL inhibits virus replication *in vivo* remain unclear. As a viricide, further mechanistic research on GL is needed.

An *in vitro* test demonstrated that GL was the most efficient and nontoxic broad-spectrum anti-coronavirus molecule, especially against SARS-CoV-2, through the disruption of the S-RBD and ACE2 interaction (Yu et al., 2021). *In vitro* studies have also shown that GL perfusion can significantly reduce adhesion between polymorph nuclear leukocytes (PMNs) and rat brain capillary endothelial cells (CCEs), which are significantly affected by herpes simplex virus (HSV) infection. This result indicates that GL may reduce the inflammatory response induced by HSV through inhibiting the adhesion of CCEs and PMNs (Huang et al., 2012).

#### Anti-inflammatory activity

Takei et al. (2006) evaluated the anti-inflammatory activity of GL through a systemic inflammatory response syndrome (SIRS) mouse model. Studies have shown that GL can inhibit SIRS by inhibiting polymorph-nuclear neutrophils (PMNs) from producing CC-chemokine ligand 2 (CCL2). The working mechanism of GL against inflammation is regulated through the NFκB signaling pathway. Previous reports indicate that both GL and its aglycone have mineralocorticoid functions due to their inhibition of liver D′-5-β-reductase (Gallacher et al., 2017). GL also exhibited anti-arthritis function through increased hydrocortisone production. GL and its aglycone (glycyrrhetic acid) can block the NF-κB/MCP-1 and MAPK/ERK/MCL-1 signaling pathways in THP-1 cells, increase cell apoptosis, and reduce systemic inflammation (Tan et al., 2018).

#### Antitumor activity

Recent studies have reported that GL and its derivatives can bind to PGRMC1 (progesterone receptor membrane component 1), inhibit the interaction between PGRMC1 and EGFR (Epidermal growth factor receptor), then inhibit HCT116 and HuH7 cancer cell proliferation, while GA does not bind to PGRMC1 (Kabe et al., 2021). GL can significantly inhibit the growth, migration and invasion of leukemia cells *in vitro* and in BALB/c mice by down-regulating AKT-mTOR signaling pathway activity and STAT3 phosphorylation (He et al., 2015). Oral administration of GL (15 mg/kg) produced a significant inhibition of 1,2-dimethylhydrazine (DMH)-induced colon cancer in Wistar rats. In addition, GL inhibited tumorigenesis by reducing hyperproliferative response, inflammation, and apoptosis (Khan et al., 2018). GL can act as a free radical scavenger, inhibiting the genotoxicity of oxidative mutagens induced by H_2_O_2_ *in vitro* (Kaur et al., 2012).

#### Antiallergic activity

In a chronic asthma mouse model, after 7 consecutive days of administration, GL significantly reduced the number of goblet cells and mast cells, as well as the thickness of the basement membrane, and it inhibited the proliferation of the subepithelial smooth muscle layer and lung epithelium. There was no significant difference in the effect between GL (10 mg/kg/d) and dexamethasone (1 mg/kg/d) administration (Hocaoglu et al., 2011).

Another report found that GL could increase the transcription level ofthe β2-adrenergic receptor in rats and increase the accumulation of cAMP *in vitro* at a dose of 0.3 µM. The combination of GL and salbutamol induced significant complementary anti-inflammatory effects. The mechanism of GL against asthma and inflammation is closely related to the regulation of the NF-κB signaling pathway (Yang et al., 2010). GL also exhibited an anti-inflammatory effect against asthma by modulating the TGFβ1-Smad signaling pathway in asthma-associated airway inflammation mice model, and then remodeled the airway (Yao and Fu, 2021). Therefore, combination therapy with GL and other antiallergic drugs exhibits synergistic effects.

#### Antihyperglycemic (diabetic) and hyperlipidemia activity

GL administration can improve streptozotocin-induced diabetes in rats. It helps improve glucose intolerant behavior by inhibiting oxidative stress and reducing serum insulin levels (Sen et al., 2011).

#### Hepatoprotective effect

In a clinical phase III study, GL showed good improvement and tolerability in patients with chronic hepatitis C who did not respond to interferon (IFN) treatment (Manns et al., 2012). Basic pharmacological studies also showed that GL could prevent hepatitis induced by concanavalin A (Con A) through inhibiting liver iNOS (nitric oxide synthase 2, inducible) induction and hepatocyte degeneration. In this study, GL inhibited iNOS transcription and translation levels and decreased AST and ALT levels induced by Con A (Tsuruoka et al., 2009). GL can also block LPS/D-galactosamine-induced liver injury by inhibiting the inflammatory response and IL-8 production in mice (Yoshida et al., 2007).

However, some side effects are caused by GL administration, including pseudoaldosteronism, which limit its clinical applications. Therefore, combination application of GL with other drugs is a good method for targeting diseases therapy. For example, the administration of matrine plus GL shows better liver protection and liver cancer suppression than GL administration alone. In addition, its combination with other traditional Chinese medicines can also reduce the adverse effects of GL, including sodium and water retention (Wan et al., 2009).

#### Neuroprotective effect

It has been reported that GL exhibits a neuroprotective effect in cerebral ischemia in middle cerebral artery occlusion (MCAO) rats by inhibiting the activation of microglia and the induction of pro-inflammatory cytokines (Luo et al., 2013). The neuroprotective effect of GL in the neuronal death model induced by Kainic acid is achieved *via* inhibiting inflammation and toxicity. GL also exhibits anti-neurotoxic activity on SH-SY5Y cells induced by 6-hydroxydopamine (6-OHDA) and corticosterone (CORT) through autophagy signaling pathway regulation (Yang et al., 2018).

GL protects neurons against oxidative stress. GL can increase the levels of SOD and NRF1 in the right hemisphere and then reduce brain edema, vacuoles, degeneration, and neuronal destruction (Akman et al., 2015). The administration of GL can also inhibit memory loss induced by AlCl_3_ and neuroinflammation in insulin-resistant rats through TLR4 signaling pathway regulation (Ali et al., 2019). GL can act as a HMGB1 inhibitor in neuroprotection. In a rat model of persistent seizures caused by lithium-pilocarpine, GL inhibited hippocampal HMGB1 expression and nuclear translocation then reduced the mortality rate (Li et al., 2019). GL can also reduce inflammation in microglia upon LPS stimulation by inhibiting the activation of the TLR4-NFκB signaling pathway (Sun et al., 2018).

#### Renal protective effect

GL can attenuate nephrotoxicity caused by an overdose of cancer drugs. According to previous reports, intraperitoneal injection of cisplatin can enhance the generation of lipid peroxidation, xanthine oxidase, and H_2_O_2_ in the kidneys of mice. Cisplatin can also reduce glutathione levels and inhibit antioxidant enzyme activity. The administration of cisplatin will induce DNA strand breakage and micronucleus formation and damage normal kidney structure. However, pretreatment with GL can help to prevent oxidative stress and reduce kidney damage caused by cisplatin (Arjumand and Sultana, 2011).

#### Thrombin inhibitory activity

GL is the first known plant-based thrombin inhibitor. It can inhibit platelet aggregation caused by thrombin activation in rats (Mendes-Silva et al., 2003). Pretreatment with GL (300 mg/kg) can reduce the average thrombus weight and anti-thrombin gene transcription levels in the liver as well as the inferior vena cava in rats (Nakata and Kira, 2016). The mechanism of how GL affects thrombin remains unclear, and additional studies are necessary.

#### Steroid hormone activity

Both GL and its metabolite GA exhibit steroid homogenization activity (Ojima et al., 1990). However, it would be better to modify their chemical structure to improve their specific organ targeting ability. More importantly, the phytoestrogen effect of GL should be considered during the application in postmenopausal women. GL can exhibit functions similar to those of glucocorticoids. In addition, GL can also reduce the affinity between the dexamethasone and glucocorticoid receptors by significantly reducing the expression of HSP90, but it cannot reduce the number of glucocorticoid receptors (Yoh et al., 2002).

### Pharmacological activities of glycyrrhetinic acid

**FIGURE 3:**
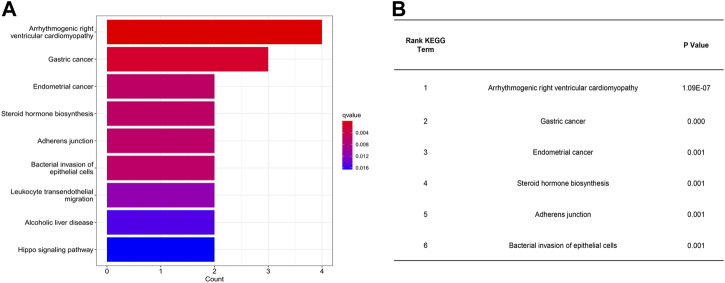
Most important interaction between 18β-GA and inflammation according to KEGG pathway analysis. **(A)** Summary of genes expressed significant differentially. **(B)** Forward 6th important enrichment signaling pathways between 18β-GA and inflammation by KEGG pathway analysis.

GL is the most important active compound in licorice. Under acidic, alkaline, or other special conditions, GL can release two molecules of glucuronic acid and one molecule of GA. Naturally, GA exhibits a β-isomer at C-18 (18β-GA). Usually the β-isomer can be isomerized into a α-isomer under alkaline conditions (Nocca et al., 2018). Importantly, the different isomers of GA exhibit different biological activities. Then we choose18β-GA for further analysis based on their OB and DL value (Table 1). Upon KEGG pathway analysis, we have found that 18β-GA shows specific targeting of diseases and signaling pathways, including arrhythmogenic right ventricular cardiomyopathy (*q* value = 3.11E-06, *p* value = 1.09E-07, *p* adjust = 4.70E-06), gastric cancer (*q* value = 0.002, *p* value = 0.000, *p* adjust = 0.002), endometrial cancer (*q* value = 0.006, *p* value = 0.001, *p* adjust = 0.009), steroid hormone biosynthesis (*q* value = 0.006, *p* value = 0.001, *p* adjust = 0.009), adherens junction (*q* value = 0.006, *p* value = 0.001, *p* adjust = 0.009), bacterial invasion of epithelial cells (*q* value = 0.006, *p* value = 0.001, *p* adjust = 0.009), leukocyte transendothelial migration (*q* value = 0.011, *p* value = 0.003, *p* adjust = 0.017), alcoholic liver disease (*q* value = 0.015, *p* value = 0.004, *p* adjust = 0.023), and the Hippo signaling pathway (*q* value = 0.017, *p* value = 0.005, *p* adjust = 0.025) (Figure 3A). KEGG pathway analysis revealed that arrhythmogenic right ventricular cardiomyopathy is the most important disease interacting with 18β-GA. Forward 6th important enrichment signaling pathways between 18β-GA and inflammation by KEGG pathway analysis (Figure 3B).

#### Antiviral activity

18β-GA can reduce the duration of viral antigen shedding in MA104 cells infected with rotavirus *via* downregulation of Fas, FasL, caspase 3, and Bcl-2 (Wang et al., 2021). 18β-GA exhibited the strongest anti-rotavirus activity (IC_50_ = 46 µM) *in vitro*, while luteolin’s IC_50_ was 116 µM and progestin’s IC_50_ was 129 µM. 18β-GA can reduce 99% of rotavirus production by inhibiting virus replication and virus entry (Hardy et al., 2012). In addition, 18β-GA inhibits coxsackievirus A16 (CVA16) and enterovirus 71 (EV71)-induced hand foot and mouth diseases in a dose-dependent manner (Wang et al., 2013).

18β-GA exhibits strong activity against the human respiratory syncytial virus (HRSV) infection of airway epithelial cells. The selectivity index in an *in vitro* antiviral test was 17.7, which means that 18β-GA is the most active compound in water extracts of licorice (Feng Yeh et al., 2013). However, the working mechanism by which 18β-GA inhibits viral infection is still not clear, and further research is needed.

#### Anti-inflammatory activity

18β-GA has also exhibited anti-inflammatory properties in different animal models (Xiao et al., 2018; Akutagawa et al., 2019; Zhang et al., 2019). Recently, two mechanisms have been elucidated regarding the anti-inflammatory effect of 18β-GA. Firstly, it interferes with complement function and exhibits immunomodulatory activity with a steroid-like structure. 18β-GA, as an effective inhibitor of 11β-hydroxysteroid hydroxylase, can cause the accumulation of glucocorticoids and stimulate glucocorticoid receptors with anti-inflammatory properties. The oral administration of 18β-GA has been confirmed to induce this glucocorticoid response (Ishida et al., 2012). Secondly, 18β-GA can inhibit complement component C2 expression (Kroes et al., 1997). Therefore, among inflammatory diseases, especially in pulmonary inflammatory diseases, 18β-GA plus hydrocortisone can exhibit a synergistic effect (Kao et al., 2010). However, some reports indicate that 18α-GA is more active than 18β-GA in metabolic diseases associated with inflammation. The working mechanism of GA against metabolic diseases is similar to that of glucocorticoids. Because 18β-GA is the main metabolite in natural GL, it exhibits no specific difference in inhibiting 11β-HSD1 or 11β-HSD2 expression, while 18α-GA specifically inhibits 11β-HSD1 (Classen-Houben et al., 2009). GA is commonly administered orally to treat liver inflammation, especially in Asia. GA also exhibits significant inhibition on liver fibrosis induced by CCl_4_ through upregulating Nrf2 nuclear translocation and inhibiting oxidative stress at a dose of 50 mg/kg/d (Chen et al., 2013).

#### Antitumor activity

GA suppresses hepatocellular carcinoma cell proliferation by causing cell cycle arrest, apoptosis and autophagy and IRE1α downregulation *in vitro* and *in vivo* (Chen J. et al., 2018). GA also exhibits antitumor activity in mice against 12-O-tetradecanoyl phorbol-13-acetate (TPA) by inhibiting oxidative stress (Agarwal et al., 2005). GA is a promising chemopreventive and therapeutic antitumor drug due to its selective toxicity against cancer cells (Agarwal et al., 2005; Yamaguchi et al., 2010).

The administration with 18α-GA can prevent the invasion of DU-145 prostate cancer cells by downregulating the levels of NF-ĸB (p65), VEGF and MMP-9, as well as HMGB1 and IL-6 (Shetty et al., 2011). 18α-GA also shows a dose-dependent inhibition of the human leukemia HL-60 cell line through the activation of exogenous and endogenous pathways. 18α-GA can reduce mitochondrial membrane potential and increase the activity of caspase-3, -8, and -9, then inhibit cancer cell proliferation (Huang Y. C. et al., 2016). 18β-GA can also be used as a chemopreventive agent against liver cancer by inducing apoptosis and inhibiting cell proliferation *in vitro* (Hasan et al., 2016). 18β-GA can inhibit the metastasis of human gastric cancer *in vitro*, which is regulated by the TLR2 signaling pathway (Cai et al., 2018; Cao et al., 2019). GA inhibits tumor initiation and promotion in a skin tumor model of SENCAR mice induced by 7,12-dimethylbenz [α]anthracene (DMBA) and 12-O-tetradecanoylphorbol-13-acetate. However, as a tumor initiation inhibitor, 18β-GA is more effective than 18α-GA (Wang et al., 1991).

#### Antibacterial activity

18β-GA also inhibits bacterial growth. The concentrations of 18β-GA that effectively inhibit *Bacillus subtilis* and *Staphylococcus epidermidis* growth are 7.6 and 12.5 μg/ml, respectively (Huang et al., 2016). The administration of 18β-GA and antibiotics exhibits a synergistic effect. 18β-GA can enhance the effect of the aminoglycosides tobramycin, gentamicin, and amikacin as well as polymyxin B, against methicillin-resistant *Staphylococcus aureus* by 32∼64 fold (de Breij et al., 2016). Although most studies of 18β-GA are focused on combination therapy with antibiotics, there is still little evidence to illuminate which bacteria is specifically inhibited by 18β-GA. Therefore, further research evaluating the effects and mechanisms of 18β-GA against bacteria is needed. Earlier reports indicated that GA, as the main active component of licorice, was able to inhibit the mutagenicity of *Salmonella typhimurium* TA98 and TA100 induced by benzo[α] pyrene (B[α]P), 2-aminofluorene and aflatoxin B1 (Malekinejad et al., 2022). As an antimutagenic agent, 18β-GA is more effective than 18α-GA (Wang et al., 1991). More research is still necessary to determine Why did 18β-GA and 18α-GA exhibit different inhibitory effects against bacteria, and its results will be useful in finding means to overcome drug resistance in therapy for bacterial infection.

#### Antiprotozoal activity

18β-GA could cure visceral leishmaniasis completely in a BALB/c mouse model. 18β-GA exhibits anti-Leishman activity by increasing NO production and Th1 cytokine production (such as IL-12, TNF-α, and IL-1β), while reducing IL-10 and TGF-β production, which is regulated by MAP kinase-p38/ERK and MAPK-MSK1 signaling pathways (Ukil et al., 2011).

#### Antioxidant activity

Oral administration of GA can increase the enzyme activity of GSH, SOD, CAT, and GPx, then reduce the content of lipid peroxidation in lung tissues of SD rats exposed to monocrotaline (MCT) (Zhang et al., 2019). In addition, GA inhibited 12-O-tetradecanoyl phorbol-13-acetate (TPA)-induced oxidative stress *in vivo*. This is also a working mechanism of GA against inflammation and tumors (Agarwal et al., 2005).

Another study showed that 18β-GA could be used as an antihepatotoxic agent (Gumpricht et al., 2005). Preincubation of rat hepatocytes with GL or 18β-GA reduced the production of reactive oxygen species (ROS). 18β-GA shows greater scavenging ability than GL in scavenging ROS. 18β-GA reduces cytotoxicity produced by glycodeoxycholic acid (GCDC) in hepatocytes *via* preventing necrosis and apoptosis, while GL reduces cytotoxicity in hepatocytes induced by GCDC *via* increasing cell apoptosis (Gumpricht et al., 2005).

#### Antihyperglycemic (diabetic) and hyperlipidemia activity

Diabetes is related to profound changes in plasma lipid and lipoprotein profiles. The lipoprotein profile is also associated with premature atherosclerosis, insufficient coronary blood supply, and increased risk of myocardial infarction. One of the main pathogenic mechanisms of diabetes dyslipidemia is induced by fatty acids released from adipose tissue and free fatty acids in the blood (Gandhi et al., 2017). In a diabetic rat model, treatment with 18β-GA significantly inhibited the levels of total cholesterol, triglycerides, free fatty acids, and very low-density lipoprotein (VLDL) cholesterol in plasma (Kalaiarasi et al., 2009; Kalaiarasi and Pugalendi, 2009). In addition, oral administration with 18β-GA for 45 days can also lower blood glucose, decrease liver glycosylated hemoglobin (HbA1c) levels in diabetic rats, inhibit gluconeogenesis enzyme activity, and exhibit antihyperglycemic activity (Kalaiarasi and Pugalendi, 2009). However, the working mechanism of 18β-GA against diabetes is still lack of evidence. More research is necessary.

#### Hepatoprotective effect

GA makes a great contribution to clearing biological toxicity, including increasing cytochrome P450 2E1 activity and anti-oxidant enzyme activity, then clearing oxidative stress induced by CCl4 or D-galactosamine in vivo, and it exhibits a hepatoprotective effect against CCl4. After administration with GA, serum AST/ALT levels and liver MDA levels decreased significantly, and the activities of liver antioxidant enzymes such as GSH-Px, SOD, and CAT increased significantly (Chen et al., 2013). However, there is still lack of evidence in comparing the hepatoprotective effects of 18α-GA and 18β-GA. More research on their pharmacology comparison is needed.

#### Neuroprotective effect

18β-GA can inhibit the cytotoxicity of PC12 cells induced by serum/glucose deprivation and 6-hydroxydopamine (6-OHDA) through mitochondrial function and PI3K/AKT signaling pathway regulation. The preventive or therapeutic administration with 18β-GA can significantly suppress the disease severity of experimental autoimmune encephalomyelitis by inhibiting microglial activation-mediated central nervous system inflammation *via* suppression of the MAPK signaling pathway in C57BL/6 mice (Zhou et al., 2015). However, research on the mechanism how does 18β-GA exert neuroprotective effects *in vivo* is still lacking. More evidence and further research are needed.

#### Cardioprotection

18β-GA can improve ischemia‒reperfusion with anemoniasulcata toxin II-induced diastolic cardiac dysfunction in a dose-dependent manner in rats. The action mechanism of 18β-GA against diastolic cardiac dysfunction may be through inhibiting the enhanced late sodium currents (Han et al., 2020). GA can also relieve the susceptibility and incidence of fatal ventricular arrhythmia during reperfusion in rat hearts by inhibiting both the L-type calcium current and transient outward potassium current as well as prolonging the duration of the action potential (Wu et al., 2015).

#### Steroid hormone activity

GA also exhibits mineralocorticoid and androgenic properties. It can decrease cortisone production but enhance cortisol synthesis, similar to inhibiting 11β-hydroxysteroid dehydrogenase type 2 (11β-HSD-2) enzyme activities *in vitro* and *in vivo*. GA also inhibits the production of deoxycorticosterone and dehydroepiandrosterone conjugates stimulated by forskolin *in vitro* and *in vivo* (Al-Dujaili et al., 2011).

GA exhibits several different mechanisms in the regulation of steroid metabolism due to its different isomers. Firstly, 18α-GA preferentially inhibits the activity of 11β-HSD-1, while 18β-GA selectively inhibits the activity of 11β-HSD-2 (Classen-Houben et al., 2009). Secondly, GA can also inhibit the activity of cytosolic 5β-reductase and microsomal 3β-HSD enzyme *in vivo* (Latif et al., 1990). All of the functions of GA mentioned above can induce a steroid response. The cortisol activity of GA can also help it suppress inflammation and immunity *in vivo* (Cirillo et al., 2017). Evidence is still lacking to fully explain the differences in steroid hormone activity exhibited by the two isomers of GA.

## Discussion

Among its pharmacological effects, GL exhibits great inhibition of viral infection, inflammation, tumor development, allergic reaction, thrombin, and diabetes, as well as protection for the liver, neuro, and renal organs and steroid hormone activity; 18-βGA exhibits strong inhibition of viral infection, inflammation, bacterial and protozoal infection, diabetes, tumor development, and oxidative stress, as well as protection for the liver, neuro, and cardiac organs and steroid hormone activity. Comparing these effects between GL and GA (Table 3), we found that GA, as the major metabolite of GL, is the most active ingredient among licorice. GA and GL both exhibit similar characterize in their pharmacology including inflammation inhibition, tumor development inhibition, viral and bacterial infection inhibition and so on. Biochemical studies found that administration of licorice, including GL or GA *in vivo*, both could induce pseudoaldosteronism, due to the hyperactivity of the mineral corticoid receptor. The studies of Toshiaki Makino’s group proved that 18β-glycyrrhetyl-3-O-sulfate is an alternative causative agent of pseudoaldosteronism after detecting and identifying the metabolites in plasma or urine from patients prescribed with licorice or animal models administrated with licorice (Ishiuchi et al., 2019; Takahashi et al., 2019). That research reported that human or other *in vivo* models contains with sulfotransferase can make a conjugation sulfate with a hydroxyl group at C-3 and glucuronyltransferase that conjugates glucuronic acid with a carboxylic acid group at C-30 of GA (Morinaga et al., 2018). This means the chemical position of GA in C-3 and C-30 is very important for chemical structure modification for GA and GL to reduce side effects and increase compound activity. However, further research on structure-activity-relationship of GL or GA is needed, which can provide more evidence to support the drug combination and clinical application of GA analogs.

**TABLE 3:** Pharmacological activities of GL and GA.

	Glycyrrhizic acid (GL)	Glycyrrhetinic acid (GA)
Pharmacological activities	Antiviral activity	Antiviral activity
	Anti-inflammatory activity	Anti-inflammatory activity
	Antitumor activity	Antitumor activity
	Antiallergy activity	Antibacterial activity
	Antihyperglycemic (diabetic) and hyperlipidemia activity	Antiprotozoal activity
	Hepatoprotective effect	Antioxidant activity
	Neuroprotective effect	Antihyperglycemic (diabetic) and hyperlipidemia activity
	Renal protective effect	Hepatoprotective effect
	Thrombin inhibitory activity	Neuroprotective effect
	Steroid hormone activity	Steroid hormone activity
		Cytotoxic activity
		Cardioprotection

### Structure modification

GA seems to be the most active component in therapy among all the components in licorice. However, GA induces both cytotoxicity and mineralocorticoid-like effects during therapy. Therefore, there is an urgent need to modify the chemical structure of GA to increase the specific binding of GA and reduce its side effects.

Cai’s early report showed that modification of 18α-GA at the C3 and C28 positions could increase its antitumor activity *in vitro* (Cai et al., 2019). Huang’s research team also showed that the introduction of a phosphate moiety into C-28 of 18α-GA can improve its anticancer activity and enhance NF-κB nuclear translocation induced by TNF-α. The specific inhibition of NF-κB activation by 18α-GA is due to the different carbon chain lengths at C3 position. When an electron donor substituent (-OCH3) or halogen group (-F) is introduced into the 3rd position of the benzene ring, with the deacetylation effect of 3-OH on 18α-GA, the inhibitory effect of GA on NF-κB nuclear translocation decreases significantly (Jin et al., 2018b). The introduction of the aminobenzothiazole moiety into C30 position of 18β-GA can decrease the interaction between HSP90-Cdc37 complexes and 18β-GA. The introduction of the ester linking group into C3 of 18β-GA can also increase its antitumor activity and reverse drug resistance (Jin et al., 2018a).

The introduction of triphenylphosphine cations (TPP+) into C3 or C30 of 18β-GA can improve its antitumor activity (Beseda et al., 2010). Lipase A catalyzes the reaction with ethanolamine at C3 or C30 position of 18β-GA, and the resulting molecule exhibits significant antiviral activity against herpes simplex virus type 1 (HSV-1) TK + and TK- strains. Introducing a dextrin linker into C3 or C30 of GA can change its anti-influenza virus activity (Zigolo et al., 2018; Liang et al., 2019).

Another report showed that hydroxylation at C-7 or C-15 position of 18β-GA could inhibit the production of nitric oxide and increase its anti-inflammatory activity. However, modifying the position of C-3 with carbonylation can cause adverse effects (Fan et al., 2019). Modifying the A and C loops of 18β-GA enhances its anti-inflammatory and antitumor activity in ICR mice (Markov et al., 2018). The introduction of different heterocycles conjugated with α, β-unsaturated ketones into ring A of 18β-GA can increase apoptosis induction. This could be a new strategy to synthesize analogs against cancer that are based on the 18β-GA chemical structure (Alho et al., 2019).

### Perspectives

Licorice has been used as a traditional herbal medicine worldwide. Licorice has been recognized as an “excellent coordinator” because it can coordinate most herbal medicine pharmacology in clinic around China. As reported in Shang Han Za Bing Lun, licorice is frequently used in traditional prescriptions (Yang et al., 2015). As its main ingredients, GA and GL both exhibit strong effects against inflammation and tumors. They also exhibit strong pharmacological effects against viral and bacterial infection. GA and GL can protect the liver, heart, nerves and kidney from inflammation.

In most researchers’ opinion, discovering specific binding sites with known functional proteins is a productive means of discovering new, more active, and lower-toxicity compounds against diseases, based on the chemical structure of GA or GL. High-throughput screening through chemical structure modification is the most efficient way to obtain analogs. However, most chemical compounds with isomers still exhibit nonspecific binding toward proteins or tissues. For GA and GL, the most common side effects are excessive mineralocorticoids, manifested as sodium retention, hypokalemia and hypertension, which are caused by their nonspecific binding (Yang et al., 2015). Special attention should be paid to these compounds when using parent compounds or analogs in clinical application.

In this article, we collected information on the pharmacology of GA and GL as well as their known structural-activity relationships. Licorice is a well-established herbal medicine that has been used in clinics for a long time. GA and GL, the major components of licorice, can both inhibit inflammation induced by viral or bacterial infection, and effectively protect the liver, kidney, heart, and nerves. However, GA has been abandoned due to its cytotoxicity and side effects, such as mineralocorticoid-like super effects. The most important and efficient way to expand the application of GA and GL in clinic is to modify their chemical structure to decrease their side effects and increase their pharmacological effects during therapy on a specific disease. We have concluded that modification of the C3, C7, C15, C28, and C30 positions in GA and GL can change their activity and binding specificity. Researchers should choose the correct method and position for GA and GL as well as their analog chemical structure modification during the actual research.

## Data Availability

The original contributions presented in the study are included in the article/supplementary material, and further inquiries can be directed to the corresponding authors.
